# Preliminary Evaluation of a Brief Web and Mobile Phone Intervention for Men With Depression: Men’s Positive Coping Strategies and Associated Depression, Resilience, and Work and Social Functioning

**DOI:** 10.2196/mental.7769

**Published:** 2017-08-10

**Authors:** Andrea Susan Fogarty, Judy Proudfoot, Erin Louise Whittle, Janine Clarke, Michael J Player, Helen Christensen, Kay Wilhelm

**Affiliations:** ^1^ Black Dog Institute UNSW Randwick Australia; ^2^ St Vincent's Hospital Faces in the Street Darlinghurst Australia

**Keywords:** depression, eHealth, men, mental health

## Abstract

**Background:**

Previous research has identified that men experiencing depression do not always access appropriate health services. Web-based interventions represent an alternative treatment option for men, are effective in reducing anxiety and depression, and have potential for wide dissemination. However, men do not access Web-based programs at the same rate as women. Programs with content explicitly tailored to men’s mental health needs are required.

**Objective:**

This study evaluated the applicability of Man Central, a new Web and mobile phone intervention for men with depression. The impact of the use of Man Central on depression, resilience, and work and social functioning was assessed.

**Methods:**

A recruitment flier was distributed via social media, email networks, newsletters, research registers, and partner organizations. A single-group, repeated measures design was used. The primary outcome was symptoms of depression. Secondary outcomes included externalizing symptoms, resilience, and work and social functioning. Man Central comprises regular mood, symptom, and behavior monitoring, combined with three 15-min interactive sessions. Clinical features are grounded in cognitive behavior therapy and problem-solving therapy. A distinguishing feature is the incorporation of positive strategies identified by men as useful in preventing and managing depression. Participants were directed to use Man Central for a period of 4 weeks. Linear mixed modeling with intention-to-treat analysis assessed associations between the intervention and the primary and secondary outcomes.

**Results:**

A total of 144 men aged between 18 and 68 years and with at least mild depression enrolled in the study. The symptoms most often monitored by men included motivation (471 instances), depression (399), sleep (323), anxiety (316), and stress (262). Reminders were scheduled by 60.4% (87/144). Significant improvements were observed in depression symptoms (*P*<.001, *d*=0.68), depression risk, and externalizing symptoms (*P*<.001, *d*=0.88) and work and social functioning (*P*<.001, *d*=0.78). No change was observed in measures of resilience. Participants reported satisfaction with the program, with a majority saying that it was easy (42/51, 82%) and convenient (41/51, 80%) to use. Study attrition was high; 27.1% (39/144) and 8.3% (12/144) of the participants provided complete follow-up data and partial follow-up data, respectively, whereas the majority (93/144, 64.6%) did not complete follow-up measures.

**Conclusions:**

This preliminary evaluation demonstrated the potential of using electronic health (eHealth) tools to deliver self-management strategies to men with depressive symptoms. Man Central may meet the treatment needs of a subgroup of depressed men who are willing to engage with an e-mental health program. With further research, it may provide an acceptable option to those unwilling or unable to access traditional mental health services. Given the limitations of the study design, prospective studies are required, using controlled designs to further elucidate the effect of the program over time.

## Introduction

Global prevalence estimates show that a substantial number of men are affected by depression [1,2]. Although women are diagnosed with depression more frequently [3,4], recent research acknowledges that men may express depression differently than women [5], with some pointing to higher rates of substance use disorders, relationship problems, and externalizing behaviors among men as evidence of “masked” depression [6]. Globally, prevalence estimates of depression in men vary between 3.8% and 6.4%, depending on the measurement and definition [2]; in 2012, the rate of male suicide (15 per 100,000) was nearly double the rate in women (8 per 100,000; [7]). In Australia, the estimated 12-month prevalence of mental or substance use disorders among men is 20.4% [8], and men die from suicide at nearly 4 times the rate of women [9].

At the same time, men are less likely to seek professional help for health problems; a recent review concluded that men’s individual level of adherence to masculine role norms can significantly affect their help-seeking behavior and symptom management [10]. For some, beliefs about traditional masculinity can contribute to inhibiting emotions and perceived need for help [11]. Furthermore, the use of maladaptive coping strategies [12] is implicated in men’s risk of attempting suicide [13].

Globally, there are calls for improved longitudinal data regarding changes in the rates of men’s help-seeking behavior for mental health [14]. Whereas the proportion of Australian men seeking professional help for mental health and substance use problems increased significantly between 2006 and 2012 [15], men still access treatment at lower rates than women, and common mental health problems in men persist. Indeed, even when men see a health professional, they may not always disclose psychological distress [16]. Furthermore, men with more severe depression report higher perceived barriers to help-seeking [17], and there are persistent barriers to successful detection and management of depression [16,18]. For example, general practitioners (GPs) cite not having sufficient training in mental health and a lack of confidence in managing symptoms as specific barriers [19]. Thus, while access to services is improving, there are still men at risk of depression and suicide, who are unable to access appropriate care in a timely manner.

In recent years, the use of Web and mobile phone–based interventions has received considerable research attention [20]. Web-based cognitive behavioral therapy (CBT) programs targeting depression and anxiety are effective in reducing symptoms [21,22], are cost effective [23], have comparable treatment effects with face-to-face therapy [24], and can contribute to improvements in work and social functioning [25]. A systematic review concluded that mental health programs delivered via mobile phones could deliver reductions in depression, stress, and substance use, with the potential to improve treatment accessibility [26]. As a result, there are growing calls for the integration of effective e-mental health services with primary care, clinical, and community settings [27,28], with an emphasis on the importance of research rigor [26], the need for user involvement in the development stage [29,30], and an understanding of consumer preferences [31,32]. Research indicates that, on the whole, men do not take up Web-based interventions at the same rate as women [33], though reasons for this are not always clear. However, one study reported higher rates of acceptance of a Web-based intervention among young men than young women when the intervention was accessed via Internet from the home (as opposed to school; [34]). Thus, the question that arises is whether Web-based programs can be developed that target men and will be liked and used by men.

In this context, this research developed a brief intervention for men with at least mild depression following participatory-based design recommendations [35]. Men from around Australia were involved in two phases of research to identify the most effective self-care strategies they used to prevent and manage depression [36-38]. The results were used to develop and test a Web and mobile phone brief intervention for men with depression, in response to concerns that at-risk men in the community may not access traditional services in a timely manner. This study aimed to assess the feasibility of the intervention and to evaluate its relationship with symptoms of depression, resilience, and work and social functioning.

## Methods

### Recruitment

The study was promoted via the lead institute’s professional and digital networks. The Black Dog Institute is a translational research center that incorporates research, clinical services, and community education and focuses on achieving reductions in the incidence of mental illness and suicide rates in Australia. The study was promoted as a “Research Study: ‘Man Central’—an online tool for depression,” and the recruitment flier worded potential participation as follows: “We will ask you to visit the study website and answer a few questions to see if the study is suitable for you. If you are enrolled in the study, you will be asked to complete initial study questionnaires and complete your registration with the program. You will then be asked to use the online program for a period of 4 weeks, while tracking your moods. At the end of 4 weeks, you will be asked to complete the second round of study questionnaires.” Recruitment activities included circulation of a printed recruitment flier, social media publicity using Facebook and Twitter and links to the study on the lead institute’s websites, email circulation of an electronic flier through the organizational networks of all the partners on the study, and an invitation email to men who had previously registered to the lead institute’s volunteer research register. An email flier was also circulated via Mensheds Australia [39]. The promotional flier invited Australian men to visit the study website and register their interest in participating. This expression of interest was open between May and October 2014, and the study remained listed on the Black Dog Institute’s website under “research opportunities” during this period. Once the development of the tool was complete, all men who had registered an expression of interest in knowing more about the study were contacted via their email address and invited to visit the study website where they could participate in screening procedures to assess their eligibility. At this time, the study was publicized again via the lead institute’s social media pages with a link to the study website.

Men were eligible to participate if they scored at least 5 or more on the Patient Health Questionnaire-9 (PHQ-9; ie, mild depression), had a valid email address, could access the Internet via both a computer and a mobile phone, were aged 18 years or more, were resident in Australia, and were comfortable reading and writing English. Men were excluded on the following basis: no current depression or depression symptoms below the study threshold (PHQ-9 total score less than 5), frequent suicidal thinking (a score of 3 on item ix of the PHQ-9: “Thoughts that you would be better off dead or of hurting yourself in some way”), and/or psychosis (score of 2 or more on the Psychosis Screening Questionnaire).

### Intervention

The Man Central intervention was delivered via an existing Web-based program, myCompass [25]. myCompass was developed by the Black Dog Institute and is fully automated and delivers a personalized intervention based on the assessment of user’s symptoms at registration. Recommendations include a set of interactive, skill-based psychoeducational modules and cognitive/behavioral factors for self-monitoring. There is flexibility for users to select their own modules and self-monitoring dimensions if they wish. Additionally, access to a range of other resources, including self-monitoring reminders, mental health care tips and motivational statements delivered by email/short message service (SMS), a Web-based journal, and graphical reporting of self-monitoring data is provided.

**Figure 1:**
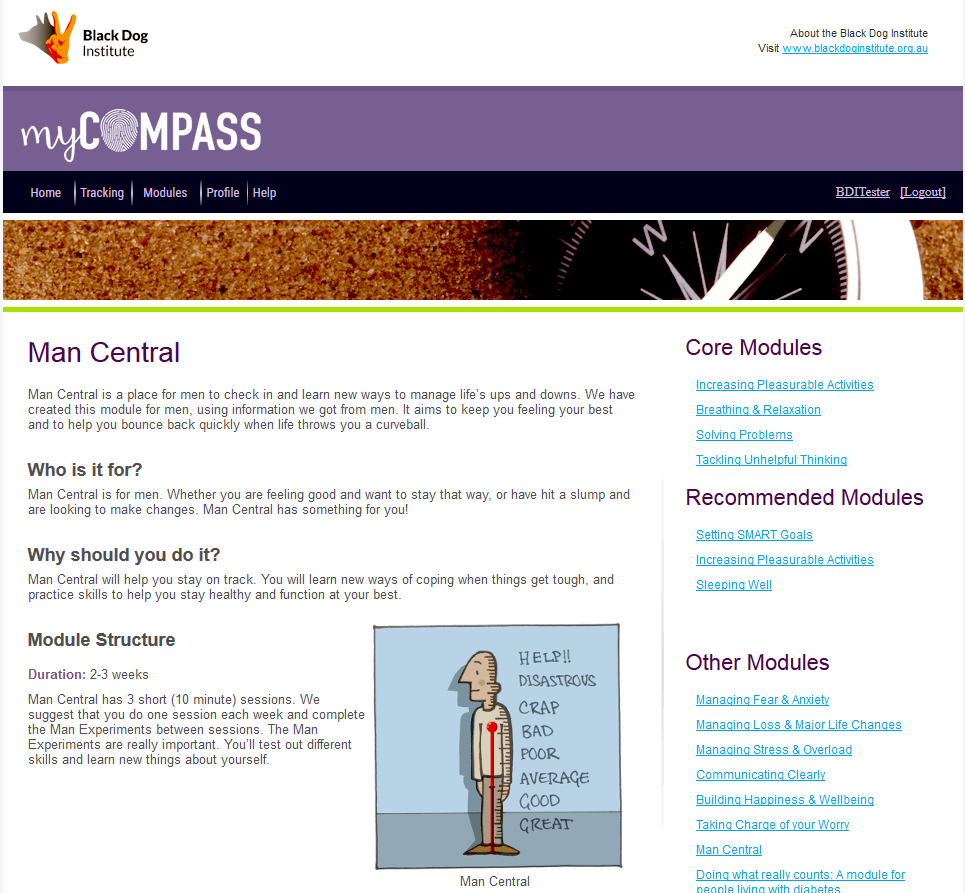
Home page of Man Central module on myCompass program.

There are currently 12 myCompass modules, including four core modules (eg, tackling unhelpful thinking), three recommended modules (eg, sleeping well), and six further modules that can be completed at the user’s convenience (eg, communicating clearly and managing fear and anxiety). For this study, access to the existing 12 myCompass modules was restricted to ensure users’ engagement with the new module (Man Central) only (see Figure 1). Man Central was developed for men with depression, based on the results from two previous phases of research exploring men’s use of and preferred positive strategies to prevent and manage symptoms of depression, including suicidal ideation [36,37].

In line with the existing myCompass modules, Man Central is delivered without therapist support, although the content was developed by a clinical psychologist in close coordination with the research team (AF and EW), followed by a review by a senior clinical researcher (JC) and project leads (JP and KW). Principles of both CBT and problem-solving therapy were used to inform the development of the module exercises, with emphasis given to the words and examples used by men in the development phase of research.

**Figure 2:**
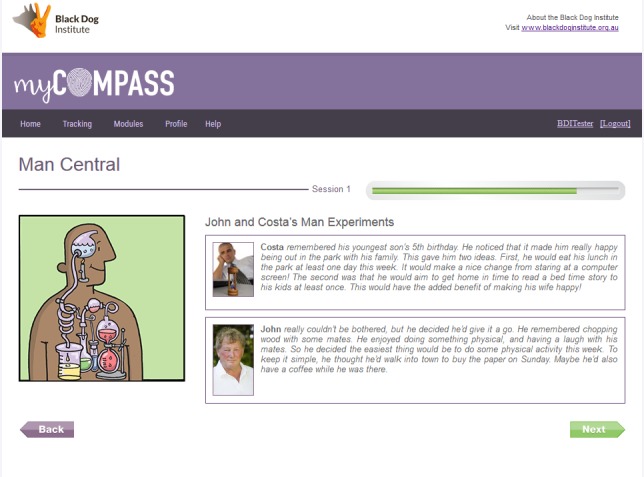
Example of “Man Experiment” with case studies.

The module comprises three brief interactive sessions and two home tasks (“Man Experiments”) to facilitate skill generalization (Figure 2). Session 1 focuses on understanding the links between moods and behavior, as well as recognizing warning signs or changes in mood. Session 2 focuses on strategies used by other men, with the user choosing some to try. Session 3 focuses on staying on track, having tried new strategies, and building a plan for the future based on what worked well.

In general, each session incorporates initial education, concept examples, a case study example, and the user applying the ideas to their personal situation by entering information into the program in response to prompts. At the end of each session, users are introduced to a homework task, where they record the activity they have chosen, plan to practice the task, and can elect to receive reminders to do the activity through the week. Upon returning for the next module session, users review their “Man Experiment” and rate their enjoyment of it. Different feedback is offered by the program, dependent on whether the experiment went better or worse than expected or whether the user felt neutral about it.

Throughout all sessions and experiments, a traffic lights analogy is used to facilitate men’s understanding of different moods and associated behaviors, which distinguishes between a Green Zone (ie, prevention), Orange Zone (ie, early intervention), and Red Zone (ie, management of a depressed mood). In addition, users learn skills regarding the identification of unhelpful strategies (ie, maintaining factors), awareness of predisposing factors and difficult situations, and choice of appropriate action. Figure 2 illustrates two case studies featuring the male characters who provide examples for men to consider when examining their own situations (Figure 2).

**Figure 3:**
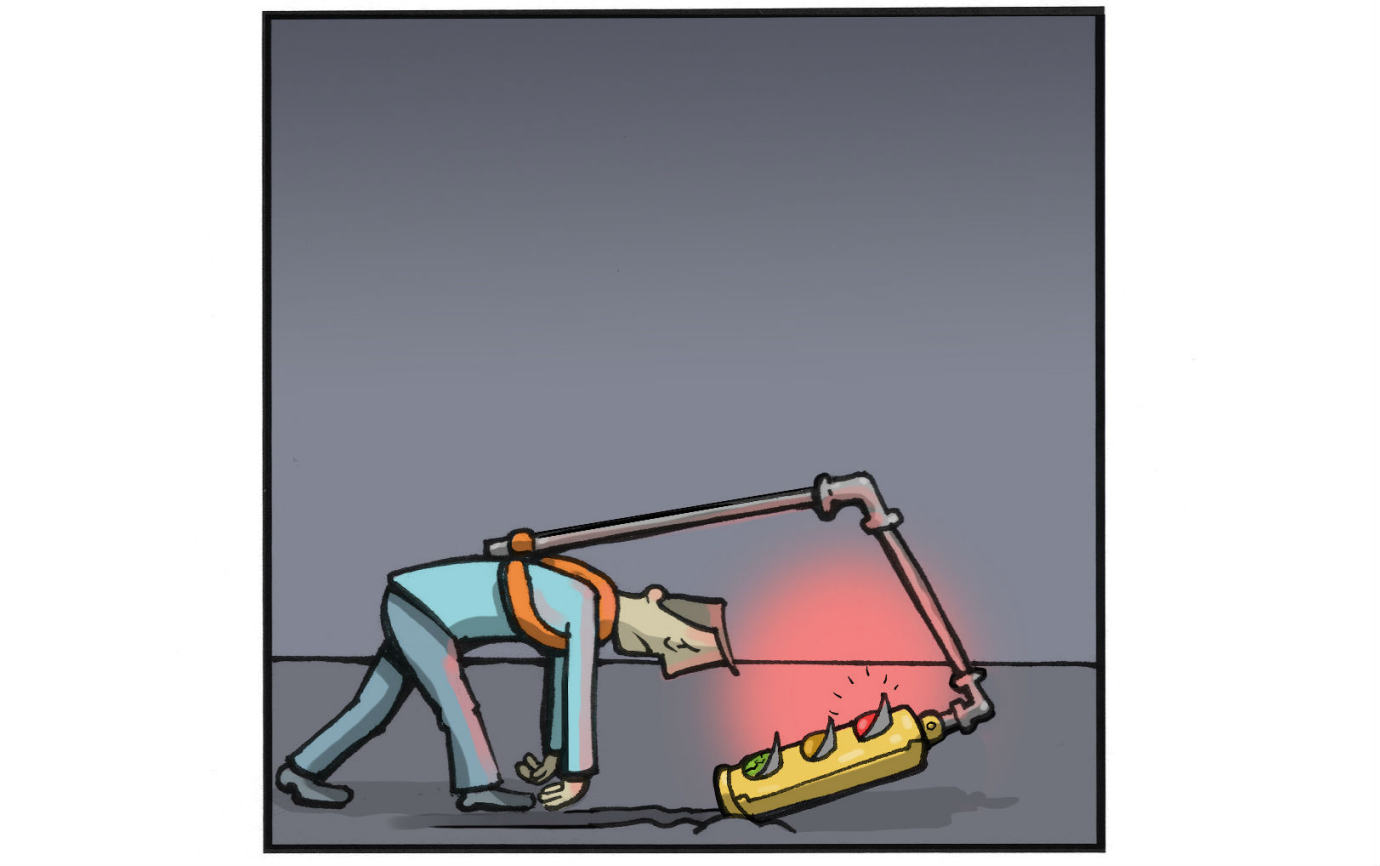
“Red Light”—traffic light imagery used in Man Central module.

The “Man Experiments” encourage men to evaluate how well those activities suited them and their lifestyle, with a view to finalizing a mental “tool kit” of strategies to be used in tough times. The module concludes with the development of a personalized “traffic lights plan,” which identifies moods, behaviors, warning signs, and appropriate helpful strategies that match a user’s Green, Orange, and Red zones (Figure 3).

In addition to completing Man Central, participants were required to perform regular “mood monitoring” using the tracking features of the myCompass program, which allowed men to monitor up to the three symptoms, moods, or behaviors, while at the same time recording contextual data (eg, where they were, who they were with, and what they were doing). Tracking was an essential feature of the intervention, given its relationship with improved outcomes [40].

### Design and Procedure

The preliminary evaluation used a single-group, repeated measures (pre-post) design to evaluate associations between the use of Man Central and the measures of depression, resilience, and work and social functioning. Interested men visited the study website to complete screening. Eligible participants who consented commenced baseline data collection immediately, followed by automated registration with myCompass.

Once enrolled in the study, users were instructed to complete mood monitoring for 4 weeks, one module session per week, and the two home tasks in between module sessions. Participants were contacted at regular intervals by the study team as per the following schedule: (1) within 3 days of registration, participants were welcomed into the study; (2) at 2 weeks, a reminder advised participants that they were at the halfway point and queried any technical problems; (3) at week 3, a reminder encouraged participants to complete the module; (4) at week 4, a reminder prompted participants to provide follow-up data; and (5) a final reminder advised participants that the study was complete and prompted follow-up data collection for anyone who had not completed the questionnaires. This was done based on previous research, which found that automated reminders could improve course-completion rates [41]. However, it should be noted that none of the contacts with the research team contained therapeutic information or support.

All data were collected online via a study-specific website, which was integrated with the myCompass program. Participants were compensated Aus $50 for their time, and access to the other myCompass modules was restored at the close of the study.

The study was approved by the UNSW Australia’s Human Research Ethics Committee (HREC13077).

### Measures

Participants provided standard demographic data (age, gender, relationship status, and location) at enrollment. Outcome measures were collected at baseline and follow-up, 4 weeks later. The primary outcome was symptoms of depression, as measured by the well-validated PHQ-9 [42-44], which asks how often a person has been bothered by particular symptoms in the previous 2 weeks. Total scores range from 0 to 27, with clinically significant cut points indicating mild (5-9), moderate (10-14), moderately severe (15-19), or severe (20+) depression.

Secondary outcomes included the assessment of externalizing symptoms of distress using the Male Depression Risk Scale (MDRS; [45]), which uses 22 items rated on an 8-point scale (0-7), with higher total scores indicating higher distress, and validated subscale scores are produced for emotional suppression, drug use, alcohol use, anger and aggression, somatic symptoms, and risk-taking. Functional impairments in work, home, leisure, and social activities were assessed via the Work and Social Adjustment Scale (WSAS; [46]). Participants rate 5 items on a 9-point scale (0-8) that generates a total score between 0 and 40, with higher scores indicating more severe impairment. The Connor-Davidson Resilience Scale (CD-RISC; [47]), which comprises 25 items rated on a 5-point scale (0-4), was used to assess psychological resilience. Total scores range between 0 and 100, with higher scores indicating higher resilience.

At follow-up, participants were also asked to rate their satisfaction with the myCompass program. Use of the intervention was monitored via the number of log-ins, symptoms monitored, and reminders sent.

### Data Analysis

All data were analyzed using the Statistical Package for Social Sciences (SPSS) version 22.0 (IBM Corp; [48]). Descriptive statistics (mean, standard deviation [SD], and range) were obtained for all baseline data. Independent *t*-tests, chi-square tests, and bivariate and point biserial correlations were used to examine relationships between demographic data and primary and secondary outcomes and differences between those who completed or dropped out of the study. Changes in the primary and secondary outcome measures between baseline and follow-up were examined using linear mixed modeling analyses [49-51], which allows for the inclusion of cases with incomplete data, with centered baseline scores on primary and secondary outcomes entered into the model as covariates. Parameter estimates were obtained using the restricted maximum likelihood method (REML), Cohen *d* was used to estimate within-group effect size, and separate analyses estimated significant changes between baseline and follow-up at *P*<.05 level.

## Results

### Participants

In total, 254 men participated in screening, and 144 met eligibility criteria, enrolled in the trial, and completed baseline data collection. Participants were excluded because of incomplete screening questionnaires (41/254; 16.1%), reporting either no symptoms of depression or symptoms that fell below the study threshold for inclusion (32/254; 12.6%), not having access to the Internet via both mobile phone and computer (14/254; 5.5%), symptoms of psychosis (12/254; 4.7%), previous use of myCompass (6/254; 2.4%), being nonresident in Australia (3/254; 1.2%), and being female (1/254; 0.4%).

Table 1 shows the demographic and clinical features of the sample. Participants were aged between 18 and 68 years, and about one-third were married (53/144; 36.8%) or single (44/144; 30.6%). The majority (118/144; 81.9%) were located on Australia’s Eastern seaboard. Just over half of the participants reported mild to moderate depression (82/144; 56.9%). With the exception of the MDRS emotion suppression subscale (mean: 20.3, SD: 4.5), where mean scores were on the higher end of the range, all MDRS subscale mean scores were below the midpoint. Mean scores on the CD-RISC (mean: 52.3, SD: 12.7) were at the midpoint of the scale, indicating midrange resilience. Participants’ mean score on the WSAS (mean: 21.4, SD: 8.0) indicated some functional impairment. The majority (87/144; 60.4%) indicated that they found it “somewhat difficult” to do their work, take care of things at home, or get along with other people. Just over one-third (51/144; 35.4%) reported that it was “very” or “extremely difficult.” Nearly half (70/144; 48.6%) of all participants reported that their ability to lead a normal life was impaired “markedly or very severely.”

**Table 1:** Sample characteristics.

Participant characteristics		(N=144)
**Age in years, mean (SD)**		40.47 (10.9)
**Age group in years, n (%)**		
	18-24	10 (6.9)
	25-34	34 (23.6)
	35-44	50 (34.7)
	45-54	35 (24.3)
	55+	15 (10.4)
**Marital status, n (%)**		
	Single	44 (30.6)
	De facto	26 (18.1)
	Married	53 (36.8)
	Divorced	21 (14.6)
**PHQ-9^a^depression severity, n (%)**		
	Mild	33 (22.9)
	Moderate	49 (34.0)
	Moderately severe	43 (29.9)
	Severe	19 (13.2)
**MDRS^b^, mean (SD)**		
	Total	61.9 (22.7)
	Emotion suppression	20.3 (4.5)
	Drug use	3.1 (5.9)
	Alcohol use	9.8 (9.7)
	Anger and aggression	11.8 (7.5)
	Somatic symptoms	10.3 (6.7)
	Risk-taking	6.7 (5.0)
CD-RISC^c^ total, mean (SD)		52.3 (12.7)
WSAS^d^ total, mean (SD)		21.4 (8.0)

^a^PHQ-9: Patient Health Questionnaire-9.

^b^MDRS: Male Depression Risk Scale.

^c^CD-RISC: Connor-Davidson Resilience Scale.

^d^WSAS: Work and Social Adjustment Scale.

### Covariates and Study Attrition

Being in a relationship at baseline was weakly correlated with lower PHQ-9 scores (*r*^2^=−.19, *P*=.02), higher CD-RISC scores (*r*^2^=.238, *P*=.004), and lower total MDRS scores (*r*^2^=−.278, *P*=.001). There were no other potential covariates identified.

There were no significant differences between those who provided complete follow-up data (39/144; 27.1%) and those who did not (105/144; 72.9%) on demographic factors or baseline measures of depression (PHQ-9, MDRS), resilience (CD-RISC), and functional impairment (WSAS).

### Postintervention Study Outcomes

Table 2 shows mean scores on the outcome measures at baseline and follow-up, and results of the linear mixed modeling analyses. With the exception of CD-RISC scores, which remained unchanged, improvement was observed for all primary and secondary outcome measures, including depression (PHQ-9), work and social functioning (WSAS), depression risk (MDRS total), and all the subscales of externalizing symptoms of depression on the MDRS (emotion suppression, drug use, alcohol use, anger and aggression, risk-taking, and somatic symptoms). Also, shown in the last column are the within-group effect sizes at follow-up for all outcomes measures. Effect sizes are all within the moderate range, with the exception of the effect size for total MDRS score, which is in the high range [52].

### Program Satisfaction and User Engagement

One-third of respondents (51/144; 35.4%) provided ratings of their satisfaction with the myCompass program post intervention. Of those, a majority reported that the program was easy to use (42/51; 82%), convenient to use (41/51; 80%), and easy to understand (35/51; 69%). Lower proportions agreed that the program kept their attention (28/51; 55%); improved their stress, low mood, or anxiety (24/51; 47%); or taught them skills to handle future problems (28/51; 55%). In general, participants reported that they found the tracking functions the most useful (eg, “tracking of moods gave me a comparison of how I was going, in a snapshot”), whereas the least useful parts of the program concerned difficulties with logging in (eg, “complex password to login in via mobile” or “login never worked”) or a lack of clarity regarding how to use the module (eg, “too much info was a little overwhelming” or “understanding some of the instructions”).

The majority of the participants (102/144; 70.8%) used the mood-monitoring features at least once and accessed the Man Central module at least once (93/144; 64.6%) during the 4-week period. The mean number of log-ins reported was 12 (SD: 15.8, range: 0-108), with 16.7% (24/144) logging in more than 20 times. The symptoms most often monitored by men included motivation (471 times), depression (399 times), sleep (323 times), anxiety (316 times), and stress (262 times). Reminders to monitor symptoms were scheduled by 60.4% (87/144), with an almost even split between email and SMS reminders.

There was no difference at baseline between men who did and did not log in to use Man Central during the intervention period, and the total number of log-ins to the program did not correlate with any primary or secondary outcome data obtained at baseline.

**Table 2:** Results of linear mixed modeling analyses: observed scores on primary and secondary outcome measures, and within-group effect sizes (Cohen *d*) post intervention.

Primary and secondary outcome measures of symptoms and functioning	Estimated marginal means		Test of fixed effects			Post intervention
	Baseline	Follow-up	Degrees of freedom	*F* statistic	*P* value	Cohen *d*
	Mean (standard error)	Mean (standard error)				
PHQ-9^a^	13.75 (0.18)	10.49 (0.35)	1,165	66.9	<.001	0.68
CD-RISC^b^	52.18 (0.54)	53.29 (1.0)	1,162	0.916	.34	−0.05
WSAS^c^	21.20 (0.28)	16.88 (0.55)	1,164	48.3	<.001	0.78
MDRS^d^ total	62.1 (0.65)	46.56 (1.3)	1,165	117.7	<.001	0.88
MDRS emotion suppression	20.21 (0.17)	18.39 (0.32)	1,161	25.1	<.001	0.50
MDRS drug use	3.06 (0.13)	1.99 (0.24)	1,167	14.8	<.001	0.39
MDRS alcohol use	9.71 (0.23)	6.63 (0.43)	1,166	38.34	<.001	0.56
MDRS somatic	10.42 (0.26)	6.78 (0.50)	1,166	39.24	<.001	0.43
MDRS anger and aggression	11.91 (0.24)	8.28 (0.46)	1,164	47.77	<.001	0.50
MDRS risk-taking	6.78 (0.16)	4.54 (0.30)	1,164	43.63	<.001	0.43

^a^PHQ-9: Patient Health Questionnaire-9.

^b^CD-RISC: Connor-Davidson Resilience Scale.

^c^WSAS: Work and Social Adjustment Scale.

^d^MDRS: Male Depression Risk Scale.

## Discussion

### Principal Findings and Comparison With Prior Work

Symptoms of depression were significantly reduced among men with at least mild depression, after using the newly developed module, Man Central, via the myCompass program for 4 weeks. The effect size was moderate (*d*=0.68), indicating that Man Central may have a clinically relevant effect for depression symptoms among men with at least mild depression. A significant reduction was also observed for depression risk *(d*=0.88), alongside improvement in work and social functioning (*d*=0.78). These findings are concordant with previous research showing that brief Web-based interventions can improve depressive symptoms and reduce risk [23,25,53] and suggest that Man Central holds promise as a self-help tool for a subgroup of men with depression who engage with the program.

Man Central differs from currently available Web-based tools in that it specifically targets men, and its development benefitted from prioritizing extensive research into what men themselves say are effective strategies for preventing and managing depression. Thus, it delivers useful program content that relates directly to the users and does not solely rely on delivering standard manual-driven CBT. An initial review into men’s coping strategies for depression and suicidality identified the use of maladaptive coping strategies as a predominant theme in the literature [38], with little exploration of what “effective coping” looks like from men’s point of view. The development of Man Central sought to redress this imbalance by specifically identifying and incorporating those positive strategies that men find preferable or already use effectively [36,37], while also recognizing other research that emphasizes (1) the importance of incorporating notions of “masculinity” into CBT psychoeducation for interventions that are better received among men [54], and (2) men’s preferences for self-reliance and independent problem solving, particularly among those men who do not access clinical services early in the course of illness [13,55].

Although it is true that these positive strategies are likely to be useful for both men and women and may have similarities with previously identified strategies for coping with depression (eg, self-care through diet, exercise, and sleep hygiene; helping other people; using humor to reframe situations and negative thinking; and setting goals and completing small achievements; [56]), the design of the module refined these ideas with men’s input and with men’s acceptance specifically in mind.

Three key features of the module may enhance the likelihood of its uptake among men who are reluctant to engage with standard clinical services, which are as follows: (1) the language used in the module repeatedly emphasizes the role that other men, rather than distant “clinicians,” played in creating the examples and module content; (2) the module allows for complete self-guided learning, which may be important for those men who prefer to solve problems independently; and (3) it culminates in creating a personal, individualized step-by-step plan that can be modified based on individual experimentation. This last point is particularly important, as our prior research showed that aside from advising men to talk about their problems, the most common piece of advice given by men to other men in a similar situation was to create a specific concrete plan to follow during times of stress [36]. As such, it is likely that Man Central is acceptable to men, especially as the log-in problems and reportedly confusing instructions have since been rectified.

Notably, the largest effect size observed here related to the MDRS, where scale items explicitly reflect externalizing features of depression thought to be more specific to men’s experiences (as opposed to the more standard clinical features of the PHQ-9). Thus, given the symptom improvement observed here, delivery of Man Central via the myCompass program may represent an alternative avenue for men to access treatment and employ useful self-care in managing their mental health.

No change was observed post intervention in resilience, in contrast to previous research, suggesting positive associations between treatment, increased resilience, global improvements, and symptom reductions [47,57]. It may be that as this sample already had midrange resilience scores at baseline, the capacity for improvement in resilience was limited or that the active ingredients in the module failed to have any effect on the measures of resilience. Another possibility is that the brief format of the intervention did not provide a therapeutic “dose” large enough to facilitate improvements in resilience. One previous study [58] reported gains in resilience in a 4-week time frame, which matched the time frame used in this study. However, we note that that research prescribed 2 hours of psychoeducation per week, whereas the time prescribed per week for Man Central was significantly less than 2 hours. Alternatively, resilience promotion among men with depression may require interventions that more directly encourage specific skill development that the module content does not adequately target, especially where depression is recurrent. For example, recent work [59] theorizes that with each recurrent episode of depression, people become less resilient and more sensitive to negative effects of less powerful stressors. This work argues that the following three resilience factors should be targeted in depression management: (1) successful stress management (or “stress-recovery”); (2) flexibility (employing different approaches in different contexts to maximize chances of success); and (3) positivity (competence, positive adaptations, and positive emotions during adversity). Alongside this, three types of intervention are recommended to improve resilience among depressed populations: stress inoculation training, positivity training, and meditation. Though the intervention focused on the positive strategies men use to proactively manage their mental health, these are not necessarily analogous to the intervention types (eg, positivity training) specified by previous work as relating to resilience building in depressed populations [59]. Likewise, identified dimensions of resilience, such as self-efficacy, self-control, and persistence through setbacks [60], may not be adequately targeted by the exercises in the module.

With regard to program engagement, and in line with other myCompass studies, the most popular program feature for the men in our study was symptom tracking [40]. In particular, the symptom tracked most often by the participants was motivation. One possibility is that difficulties with motivation constitute a salient or “core” feature of the depression experience for men, with changes in motivation potentially indicative of significant changes in mood. Further work is needed to determine whether (1) active self-monitoring of motivation is especially clinically relevant for men with depressive symptoms, (2) consistently monitoring motivation levels alongside changes in mood has any predictive or treatment value for self-managing mental health, or (3) motivation levels in men should be prioritized by treating clinicians, lest they be missed by current diagnostic and screening criteria. If so, this could be incorporated into preventive activities, self-management plans, and relapse prevention strategies for men in clinical settings. Furthermore, future research into developing the program could investigate the potential to incorporate psychoeducation strategies that focus on enhancing motivation levels as an ideal place to start for men who have not engaged with health services.

### Limitations

This preliminary evaluation was exploratory in nature and therefore has considerable limitations in drawing any causal associations. First, the study’s quasi-experimental single-group design prevents attributing improved symptoms and functioning solely to the intervention. For example, it is possible that our results reflect the natural course of remission of symptoms with the passage of time. Nevertheless, reductions in depression symptoms have been reported in controlled studies of myCompass previously [25]. When looking specifically at depression symptoms or work and social adjustment, the within-group effect sizes reported for attention-control and wait-list groups in that controlled trial were comparatively smaller (*d* range=0.01-0.27) than the effect sizes reported here, so it is possible that the use of Man Central accelerates the natural time course for men.

Second, adherence to the program was low, with almost one-third of participants not accessing the Man Central module. In addition, study attrition was high. Man Central is a purely self-help program (that is, without therapist or peer support), and low rates of program adherence and high rates of dropout attrition are not uncommon in studies of unguided interventions [61,62], potentially introducing selection bias and complicating interpretation of study findings. Nevertheless, our inspection of potential biases because of these factors yielded few differences between participants who did and did not access the module and did and did not complete postintervention questionnaires. Previous studies have found that adherence can be related to disease severity [63], but this was not the case in this sample. Similarly, it has been suggested that those who do not complete Web-based interventions may have derived benefit before dropping out [64], but it is not possible to infer that from the current data. All participants were invited to give feedback on why they did or did not use the program, but very few responded and of those who did, none reported specific complaints about the content of the program.

Some research suggests that human involvement, in the form of therapist or peer support, in Web-based interventions can reduce attrition rates, particularly among those with low levels of social support [64]. Future studies might seek to determine the impact of these features on program engagement and impact among men with depression. However, given the reluctance of some men to seek support for mental health difficulties, it is crucial to balance such an approach with the privacy and anonymity afforded by the independent and automated nature of the current program design.

### Implications and Conclusions

The results indicate that men with at least mild depression are interested in the possibility of using Web-based interventions for self-management of their mental health. We initially sought to recruit only 30 men to test the program but received expressions of interest from more than 300 men. Though not all who expressed interest were eligible, it nevertheless indicates that the idea of using electronic health (eHealth) tools to monitor and manage mental health is palatable to men and that using social media publicity and email fliers to recruit men to studies does pique the interest of some men. Similarly, the present intervention seems to provide an acceptable delivery format, in that the men used the program on both their phone and their computers; logging in multiple times from different locations and making use of multiple program features, including mood monitoring, reminders, and the Man Central module itself.

Given the well-documented low uptake of face-to-face psychological services for men and the increased risk of suicide among men with untreated mood disorders [13,65], it is crucial to establish other avenues of treatment, particularly for those men who might be isolated from other sources of support in their daily lives. Whereas the study lacked the tight controls of a randomized controlled trial, our findings show that Web-based delivery of practical skills and strategies, supported by real-time symptom monitoring, is a potential solution for reaching some men.

We note, however, that it is unclear from the data presented here whether the men who would engage with a service delivered in this way represent a distinct subgroup of men, with definable demographic or clinical features, or whether the recruitment approach and intervention failed to satisfy the expectations and needs of men more generally. Thus, we suggest that controlled investigation of the impact of Man Central is warranted, with an aim to determine: (1) the active program elements and required “dose” for symptom improvement, (2) the conditions under which symptoms gains are greatest (eg, guided vs unguided Internet interventions), (3) the factors governing adherence and program engagement, (4) the possible characteristics of consumers who do or do not wish to access treatment delivered via Web-based interventions, and (5) the relative benefit of delivering tailored interventions to men, as compared with nontailored interventions targeting the same factors.

As is the case for all Web-based interventions, future research will also need to examine and establish the best way to recruit men to such services if they are to be effectively scaled up for population-wide delivery. In particular, whereas this intervention may be useful to some men, it remains to be seen how such services should be promoted. Although there are recent developments in terms of “stepped-care” approaches to service delivery that seek to incorporate the prescription of eHealth tools as a part of initial treatment plans [66], such approaches still rely upon engaging with a health service at some point. Though large proportions of men do visit a GP, establishing uptake of eHealth into primary care is still in the early stages, and this approach would not effectively address the needs of those men who are alienated from all health services. In this case, we suggest future research should examine the potential for community delivery of Web-based mental health services through organizations that may not be specific to mental health but are already engaged with men in some way, for example, MenSheds Australia [39]. Similarly, recent policy recommendations support engaging men, particularly young men, where they are already engaged in community activities (eg, sport, schools, and workplaces) and using existing digital platforms to more effectively target Web-based treatment to men who are already looking for information online [67].

Regardless of these concerns, this study shows that by incorporating men’s preferences with a particular emphasis on developing a personalized plan for the future (something men strongly advised [36]), Man Central has the strong potential to deliver a useful and promising treatment alternative for men with depression who may not access traditional mental health services. As with all Web-based interventions, it will be crucial to establish the best ways to disseminate the program to reach those populations who are most in need of assistance.

## Abbreviations

CBT: cognitive behavior therapy
CD-RISC: Connor-Davidson Resilience Scale
eHealth: electronic health
GP: general practitioner
MDRS: Male Depression Risk Scale
PHQ-9: Patient Health Questionnaire-9
REML: restricted maximum likelihood
SD: standard deviation
SMS: short message service
SPSS: Statistical Package for Social Sciences
WSAS: Work and Social Adjustment Scale
